# Pitfalls in the diagnosis of apparent homozygous mutations: two cases of *IL10RA* deficiency inflammatory bowel disease and a literature review

**DOI:** 10.1186/s13023-025-03779-0

**Published:** 2025-07-03

**Authors:** Xiu Shi, Ziqing Ye, Lai Qian, Wenhui Hu, Ye Yang, Chunmeng He, Zhiheng Huang, Bingbing Wu, Ying Huang

**Affiliations:** 1 Department of Gastroenterology, Children’s Hospital of Fudan University, 399 Wanyuan Road, Shanghai, 201102 China; 2 Key Laboratory of Birth Defects, Children’s Hospital of Fudan University, Shanghai, China; 3 Present Address: College of Medicine and Health, University of Exeter, Exeter, EX1 2LU UK

**Keywords:** *IL10RA*, Apparent homozygosity, Uniparental disomy, Gene deletion

## Abstract

**Background:**

In East Asia, *IL10RA* is the predominant pathogenic gene in patients with very early-onset inflammatory bowel disease (VEO-IBD), frequently characterised by refractory diarrhoea and severe perianal disease, resulting in elevated death rates. *IL10RA*-deficient IBD, an autosomal recessive genetic disorder, has been documented to be inherited through the conventional compound heterozygous or homozygous mutation patterns from both parents. We present two cases with apparent homozygosity resulting from distinct causes, both of which seem homozygous on the genetic map. Further investigation reveals that detecting hidden genetic patterns is essential for accurate diagnosis, genetic counselling, and disease mechanism analysis.

**Methods:**

Peripheral blood samples were collected from two patients and their parents. Whole-exome sequencing (WES), Sanger sequencing, Comparative genomic hybridisation (CGH) and SNP array, Capture-based CNV (CapCNV) analysis, and real-time quantitative PCR (qPCR) were employed to investigate the genetic mechanisms and variants within the families. Both patients were followed up, and descriptive analyses were performed to characterise their clinical phenotypes, biochemical parameters, endoscopic findings, and intestinal histopathology. Multiple databases were systematically searched to review all cases of IBD with large *IL10RA* deletions.

**Results:**

Two families underwent WES, and a homozygous mutation was identified in the *IL10RA* gene on chromosome 11 in both families. In patient 1, pedigree analysis, CGH and SNP 4 × 180 K microarray testing identified paternal uniparental diploidy (c.301C > T/11q12.3-11q25del) [upd (11) pat] on chromosome 11. Patient 2 had compound heterozygosity (c. 299 T > G/exon2_3del), comprising a point mutation and an overlapping exon deletion mutation in IL10RA, as determined using familial analysis, Sanger sequencing, CapCNV analysis, and qPCR validation. In addition, the study systematically reviewed 15 cases of IBD patients with large deletions in the *IL10RA* gene from the literature.

**Conclusions:**

This is the first description of two cases of apparent homozygosity in *IL10RA*. This report emphasizes the importance of optimizing the genetic analysis workflow, especially when there is doubt about the initial whole exome sequencing results.

## Background

Inflammatory bowel disease (IBD), including ulcerative colitis (UC), Crohn’s disease (CD) and IBD unclassified (IBDU), is a chronic immune-regulated condition of the gastrointestinal tract with heterogeneous disease course and etiology [1]. Most cases of IBD have complex genetic backgrounds, and over 300 genetic loci have been associated with IBD, each contributing a small amount to the overall risk [2]. There are, however, certain rare forms of IBD, particularly in pediatric patients, that can be classified as monogenic, which are caused by mutations in single genes like *IL10RA*, *IL10RB*, or *XIAP* genes, and often present as very early-onset inflammatory bowel disease (VEO-IBD) [3–5]. Some patients, with onset age before 6 years of age, are diagnosed as VEO-IBD [6]. Hematopoietic stem cell transplantation (HSCT) is the only curative treatment currently.

Patients with biallelic deleterious mutations in *IL10*, *IL10RA*, or *IL10RB* often present with severe colitis and perianal disease during the infantile period. Among patients from East Asia, *IL10RA*, which is located on the 11th chromosome, is the most common causal gene [7]. Many point mutations in *IL10RA* have been reported [7–9]. The missense mutation c.301C > T (p. R101RW) in exon 3 and the silencing or splicing of c.537 G > A (p. T179T) was the common variant [3]. Structural variations (SV) is defined as sequence variants > 50 bp in size comprising unbalanced copy number variants (CNVs), including deletions, duplications and insertions of fragments, as well as balanced rearrangements, such as inversions and chromosomal translocation [10]. However, literature on pathogenic *IL10RA* SVs is rare.

For autosomal recessive (AR) diseases, these conditions are more likely to involve true homozygous mutations. Although whole exome sequencing (WES) is widely used in clinical genetic diagnostics, its technical limitations can sometimes lead to misdiagnosis as traditional AR diseases with homozygous mutations, overlooking the phenomenon of apparent homozygosity. Apparent homozygosity is a phenomena in genetic sequencing when a mutation is erroneously categorised as homozygous (two identical alleles), although it may really be compound heterozygous (two distinct alleles) or indicative of another non-authentic homozygous genetic condition [11]. The primary causes encompass: allele dropout (the inability to detect one of the two alleles), heterozygous deletion (the removal of one allele concealing the mutation on the other allele), uniparental disomy (UPD) (the acquisition of two chromosome copies from a single parent without any contribution from the other), and exon deletion mutations (deletions that coincide with another pathogenic mutation) [11, 12].

At present, no apparent homozygosity has been reported in IBD patients. We present two new cases with apparent homozygosity in *IL10RA*, caused by UPD and exon deletion mutations, respectively.

## Materials and methods

### Patients and diagnostic criteria

The patients were diagnosed with IBD according to the Porto criteria [13]. Patients included received genetic testing, and mutational pathogenicity was assessed in accordance with the recommendations of the American College of Medical Genetics and Genomics (ACMG) [14]. Informed written consent for invasive procedures and genetic sequencing. This study was approved by the Ethics Committee of the Children’s Hospital of Fudan University.

### Clinical phenotypes of two patients with apparent homozygous mutations

Demographic information, clinical manifestations, laboratory indicators, endoscopic findings, and intestinal histopathology of patients were obtained through the hospital's electronic medical records system. The height or weight at the time of first admission was recorded, and the Z-score (weight-for-age or height-for-height) was calculated using Anthroplus software version 1.0.4. Patients were followed up through hospitalization, outpatient visits, or telephone calls.

### Genetic sequencing of two patients with apparent homozygous mutations

Peripheral blood samples from the proband and their parents were collected for WES through GencapTM Human Whole Exon Probe V4.0 (MyGenostics, Beijing, China), followed by validation of these findings using Sanger sequencing. Genome-wide chromosome microarray analysis was performed using an Agilent arrayCGH 4 × 180 K custom chip to detect genome-wide genomic imbalances, and data were analysed using Agilent cytogenmics software. CapCNV and qPCR was employed to assess the exon copy numbers of the target genes, with the albumin gene serving as an internal control reference (CICLAB, Beijing, China). All sequence comparisons were based on the human reference genome GRCh37.

### Literature review

A systematic review of patients with *IL10RA* with large segment deletions in IBD was conducted by searching all English-language articles in the PubMed, Web of Science, Embase, Medline, and Google Scholar databases (from inception to July 2024) utilising the following search terms: inflammatory bowel disease, Crohn's disease, ulcerative colitis, IBD, CD, UC, IL10RA with deletions. Boolean operators (AND, OR, NOT) were employed to refine or broaden the search outcomes. The search approach employed free text phrases, MeSH terminology, or Emtree to enhance search sensitivity. Titles and abstracts in the search results were read to exclude cases with unclear genetic diagnosis.

## Results

### Clinical phenotypes of two patients with apparent homozygous mutations

Patient 1: A 4-month-old boy from a non-consanguineous Chinese parent with a history of recurrent diarrhea and oral ulcers during the neonatal period presented to the gastroenterology department. His 3-year-old sibling sister was healthy. He presented hematochezia at one month, perianal ulceration with fever at two months, and perianal abscess at three months. Exclusive enteral nutrition with an amino-acid-based formula and anti-infective therapy were not effective. He also had anemia, hypoalbuminemia and leukocytosis, and elevated C-reactive protein. Lower endoscopy demonstrated ulcerations in the sigmoid and descending colon, polypoid lesions in the descending colon and mild stenosis of descending colon. Rectal biopsy pathology suggested high proliferation of mucosal lymphoid tissue (Table 1).

**Table 1:** Clinical phenotypes of two patients with apparent homozygosity in *IL10RA*

Clinical manifestation	Patient 1: c.301C > T [upd(11)pat]	Patient 2: c. 299 T > G/exon2_3del	Normal range
Onset age (days)	17	30	/
Admission age (days)	115	202	/
Weight (Z-score)	− 1.55	− 4.89	− 2 to + 2
Height (Z-score)	− 1.63	− 0.75	− 2 to + 2
Symptoms	Diarrhea, hematochezia, perianal abscess, oral ulceration, eczema, recurrent fever	Recurrent fever, diarrhea, rectoperineal and rectovaginal fistulas, oral ulceration, eczema, umbilical hernia	/
Hb (g/L)	91.00	92.00	104–143
HCT (%)	28.30	44.60	32.0–43.0
ESR (mm/h)	19.00	45.00	0–21
Alb (g/L)	33.16	22.20	39–54
PLT (*10^9/L)	587.00	348.00	191–516
CRP (mg/L)	10.00	8.00	< 8
WBC (*10^9/L)	11.88	13.97	5.50–13.60
Endoscopy	Ulcerations in the sigmoid and descending colon, pseudo-polyps in the descending colon and stenosis of descending colon	Ulcerations and pseudo-polyps in the rectus and sigmoid colon and stenosis of the sigmoid colon	/
Treatment	HSCT	Jejunostomy for colonic stenosis HSCT	/

*Hb* hemoglobin; *HCT* hematocrit; *ESR* erythrocyte sedimentation rate; *Alb* albumin; *PLT* platelet count; *CRP* C reactive protein; *WBC* white blood cell

Patient 2: A 6-month-old girl from a non-consanguineous Chinese parent with a history of recurrent fever and diarrhea during the neonatal period presented to the gastroenterology department. She also developed rectoperineal and rectovaginal fistulas, umbilical hernia, and oral ulcer. Antibiotics and parenteral nourishment in the local hospital did not alleviate her symptoms. Laboratory tests revealed anemia, leukocytosis, elevated C-reactive protein and erythrocyte sedimentation rate, and hypoalbuminemia. Colonoscopy revealed ulcerations and pseudo-polyps in the rectum and sigmoid colon and stenosis of the sigmoid colon. Pathology of small bowel biopsy suggested a significant number of lymphocytes in the mucosa (Table 1).

### Genetic sequencing of two patients with apparent homozygous mutations

**Fig. 1:**
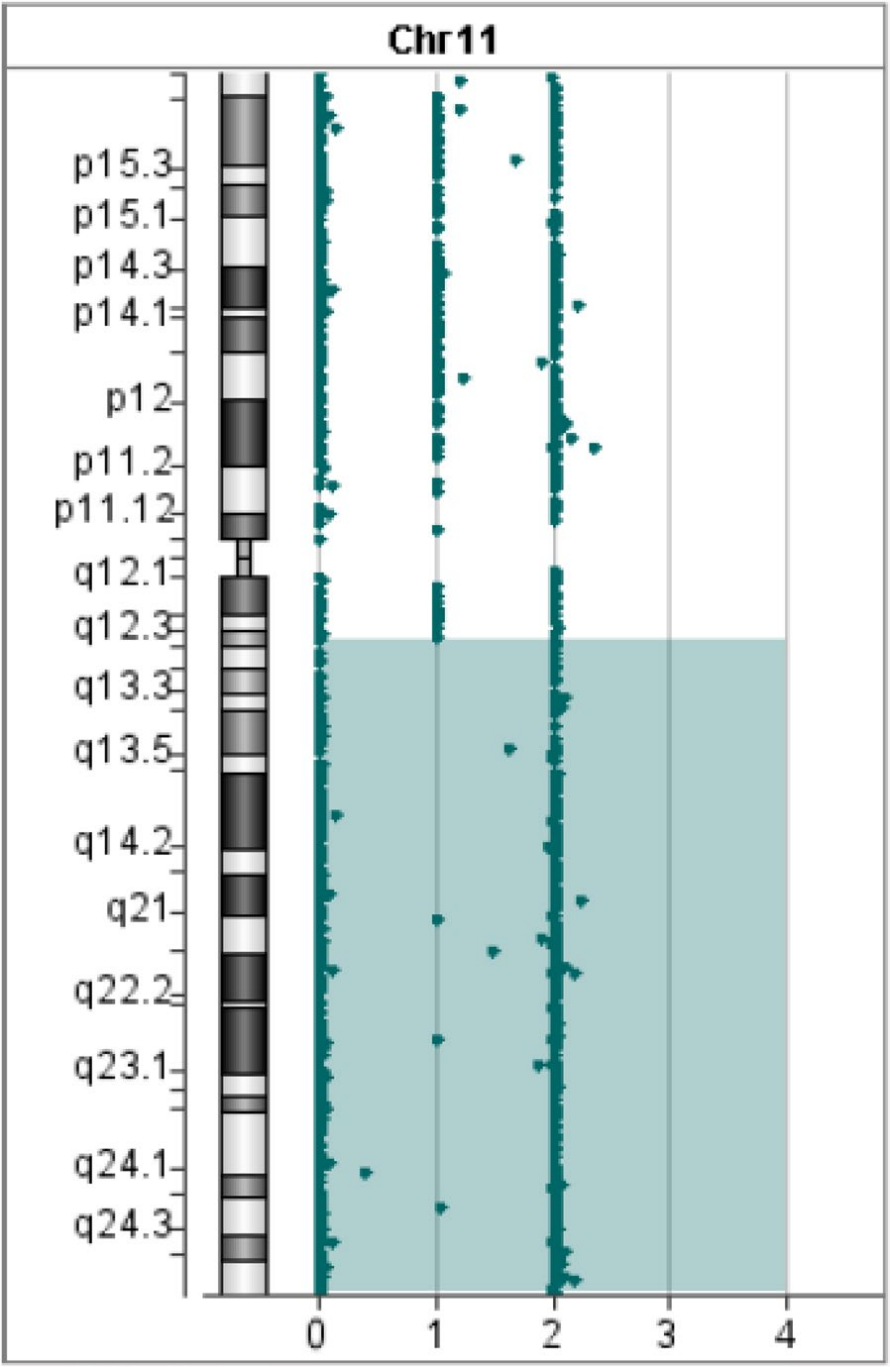
Gene microarray testing of Patient 1. The bottom number is the log2 ratios for the CGH-only microarrays

**Fig. 2:**
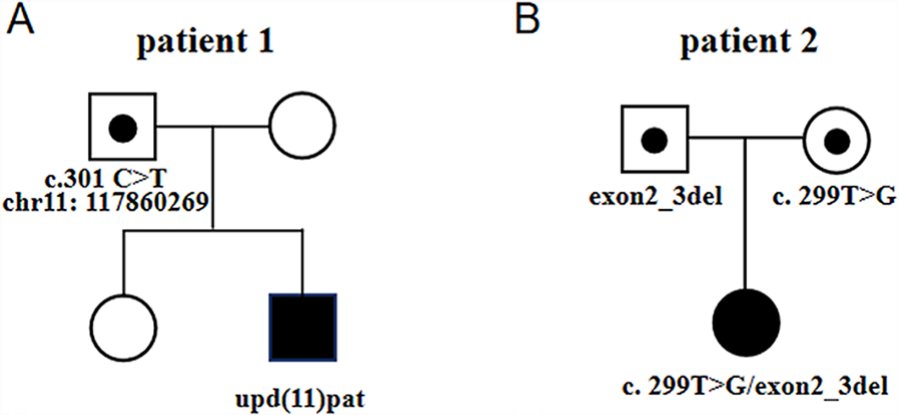
Pedigrees of the two cases. **A** Pedigree of a Chinese family with autosomal recessive *IL10RA*-deficient IBD owing to complete paternal uniparental disomy. **B** Pedigree of a patient with a large fragment loss in exons 2 and 3 at *IL10RA*. The spot represents the carrying status, while the blue filling represents the disease status

In Patient 1, WES revealed a homozygous mutation in the *IL10RA* gene (NM_001558.4: exon 3: c.301C > T chr11-117860269 (GRCh37) p.R101W) on the long arm of chromosome 11. The variant was previously reported to be pathogenic (Accession: VCV000039432.8). However, the origin of the variant suggested that the father was heterozygous and the mother had no variant, which was inconsistent with an RA mode of inheritance. Further analysis of the Agilent arrayCGH 4 × 180 K custom chip showed 0 and 2 uncut alleles, and suggested that Patient 1 had a loss of heterozygosity (LOH) in the 72.2 Mb chromosomal segment ranging from 11q12.3 to 11q25, containing *IL10RA* mapped to the area of 11q23.3 (Fig. 1). Combined pedigree analysis, clinical phenotype, and genetic analysis revealed that the disease could be caused by partial uniparental disomy [upd(11)pat] affecting the expression of imprinted genes, explaining why both variants are from the same paternal line (Fig. 2A).

**Fig. 3:**
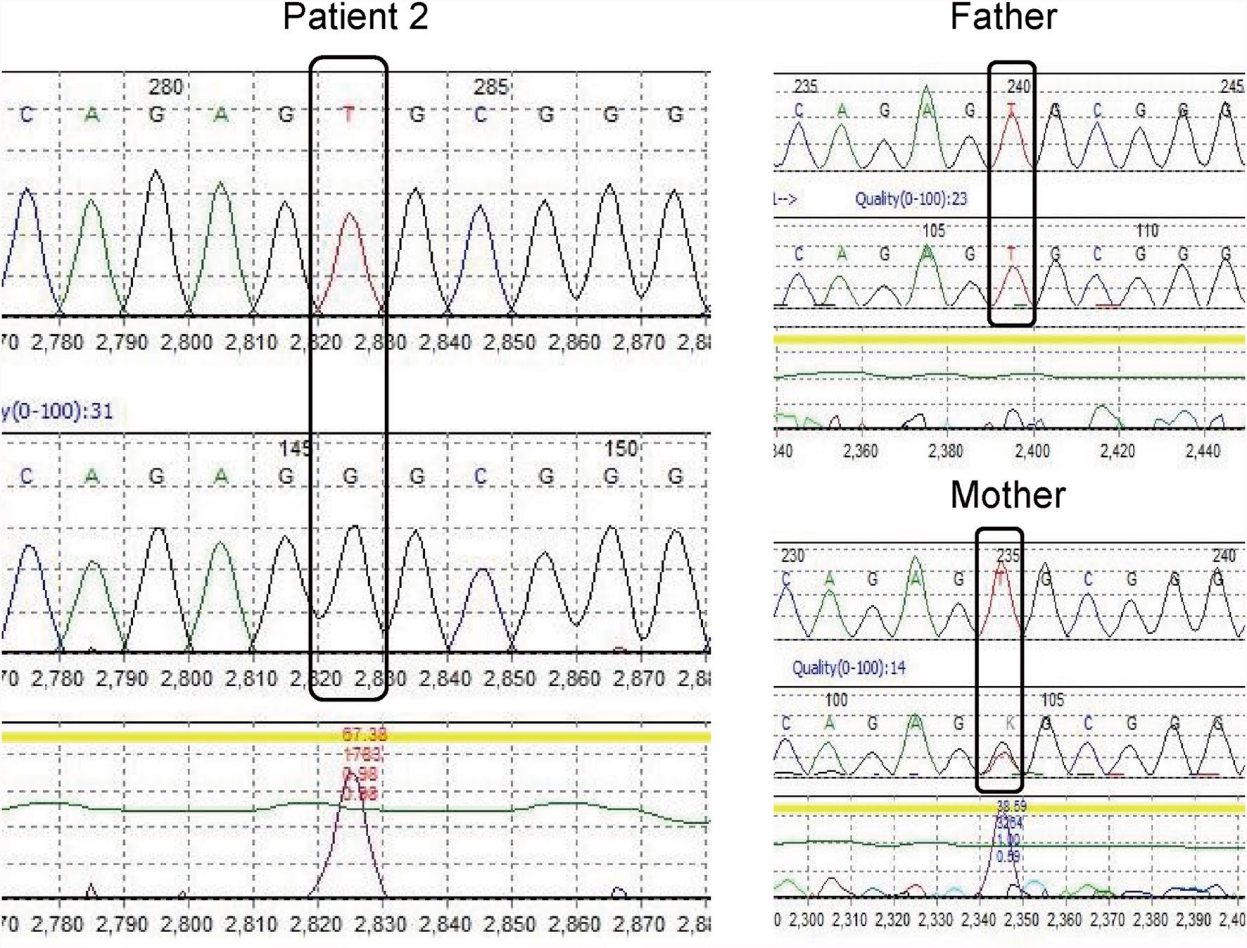
Sanger sequencing of the family of Patient 2

**Fig. 4:**
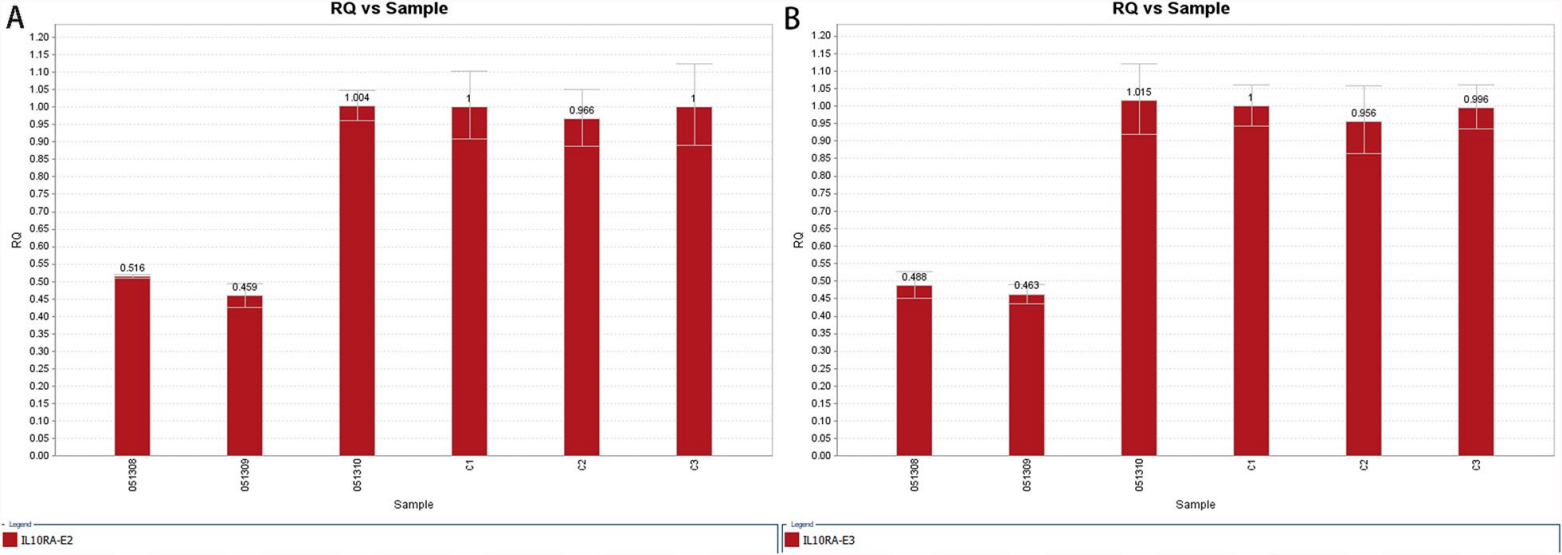
qPCR of exon 2 and exon 3 of *IL10RA* in the patient 2’s family. RQ is short for ratio relative quantity. ID 051308, ID 051309 and ID 051310 represent patient 1, the father and the mother respectively.C1, C2 and C3 belong to normal healthy people. Figure 1A shows the quantity of exon 2, while Fig. 1B shows that of exon 3. The reference gene is exon 3 of *ALB*, and relative quantity is calculated by 2^−ΔΔCt^ method

Patient 2 carried the homozygous mutation c.299 T > G in *IL10RA*, as confirmed by WES analysis. This variant has been reported before and has been proved to be deleterious [4, 15]. Sanger sequencing showed that the mother was heterozygous for *IL10RA* c.299 T > G, while the variant was not found in her father (Fig. 3). CapCNV analysis of WES data found the copy number variation in the other allele, which was a loss of large fragments spanning exons 2 and 3. Quantitative real-time quantitative polymerase chain reaction (qPCR) showed the loss of exons 2 and 3 in father and patient (Fig. 4). The point mutation and copy number variation are coincidentally located in the same area (exon 3) and were therefore initially incorrectly inferred to be homozygous mutations, whereas a combination of lineage analyses (Fig. 2B) and genetic testing ultimately determined the genotype to be a compound heterozygosity. Multiexon deletion, one type of null variant, is usually regarded as pathogenic variation according to ACMG guideline [4, 14].

### Literature review of patients with large fragment deletion in *IL10RA*

The review included 15 patients from diverse geographical and ethnic backgrounds: 12 cases from China, 1 from South Korea, 1 Arabic individual, and 1 White individual (Table 2). All parents of the patients were non-consanguineous, with the exception of case 1. 2 patients (case 1 and case 11) exhibited substantial deletions in both alleles of *IL10RA*. 13 individuals had base mutations in one allele and deletions in a substantial section of the other allele in *IL10RA*. In the majority of these individuals, the segmental deletion was situated in exon 1.

**Table 2:** Report on gene deletion of *IL10RA*

Year		Genetic mutation (paternal)	Genetic mutation (maternal)	Ethnicity	Consanguinity
2013 [16]	Case 1	Exon 1–3: exon1_3del	Exon 1–3: exon1_3del	Arabic	Y
	Case 2	Exon2:c.170A > G(p.Y57C)	Exon 2–4: exon2_4del	White	N
2021 [17]	Case 3	Exon 1: chr11:117857034–117857366 ^†^ del	Exon 3: c.301C > T (p.R101W)	Chinese	N
	Case 4	Exon 4: c.537G > A (p.T179 T)	Exon 1: chr11:117857182–117857249 del	Chinese	N
	Case 5	Exon 1: chr11:117857034–117857366 del	Exon 4: c.537G > A (p.T179 T)	Chinese	N
	Case 6	Exon 1: chr11:117857034–117857366 del	Exon 3: c.301C > T (p.R101W)	Chinese	N
	Case 7	Exon 1: chr11:117857034–117857366 del	Exon 3: c.301C > T (p.R101W)	Chinese	N
	Case 8	Exon 2: c.106G > A (p.A36T)	Exon 1: chr11:117857034–117857366 del	Chinese	N
	Case 9	Exon 4: c.537G > A (p.T179T)	Exon 1:117857034–117857366 del	Chinese	N
2021 [18]	Case 10	Exon 1: exon1 del	Exon 3: c.395 T > G (p.L132R)	Chinese	N
2021 [19]	Case 11	Exon 1: chr11:117857034–117857366 del	Exon 1: chr11:117857034–117857366 del	Chinese	N
	Case 12	Exon 1: chr11:117857034–117857366 del	Exon 4: c.537G > A (p.T179 T)	Chinese	N
	Case 13	Exon 1: chr11:117857034–117857366 del	Exon 4: c.537G > A (p.T179 T)	Chinese	N
	Case 14	Exon 1: chr11:117857034–117857366 del	Exon 3: c.301C > T (p.R101W)	Chinese	N
2023 [20]	Case 15	Exon 1: exon1 del	Exon 4: c.537G > A (p.T179 T)	Chinese	N

^†^The variants are localized according to the GRCh37.p10 reference genome

All patients had onset of disease in childhood, with 14/15 patients having onset of disease within one year of age. The primary clinical symptoms included severe diarrhoea and perianal abscesses. 8 cases were documented endoscopically in the literature as exhibiting severe colitis, colorectal ulcers, intestinal stenosis, or intestinal perforation. 5 patients were reported to have experienced bloody stools over the course of the disease, while 7 patients were noted to have anal fistulae. Alongside gastrointestinal symptoms, all patients had extraintestinal signs, including perianal lesions, recurrent fever, mouth ulcers or infections. Among the laboratory parameters, elevated White blood cell (WBC), CRP and abnormalities of lymphocytes and immune function were mentioned in some papers. Case 2 had enterostomy for intestinal perforation, whereas Case 8 received enterostomy due to severe infections and perianal lesions. 4 patients had HSCT, and 1 succumbed to post-transplant sepsis. In conclusion, all patients had an early start of illness, extensive systemic lesions, and poor prognosis, requiring the selection of appropriate treatment.

## Discussion

Homozygotic mutations could be detected via WES, while the mutation seems to be uniparental origin according to Sanger validation. In terms of Medical Genetics Laboratories at Baylor College of Medicine’ s review [11] on their 12,406 cases from 40 different genetic tests in 2012, 75 cases for whom both parental samples were available presented apparent homozygosity of point mutations or small insertions/deletions detected by PCR and Sanger sequencing. The homozygosity of 12% (9/75) of the patients could not be confirmed by parental testing. Case reports regarding this phenomenon also exist in other Mendelian diseases, such as Harlequin ichthyosis [12], cystic fibrosis [21], and mucopolysaccharidosis type VI [22].

**Fig. 5:**
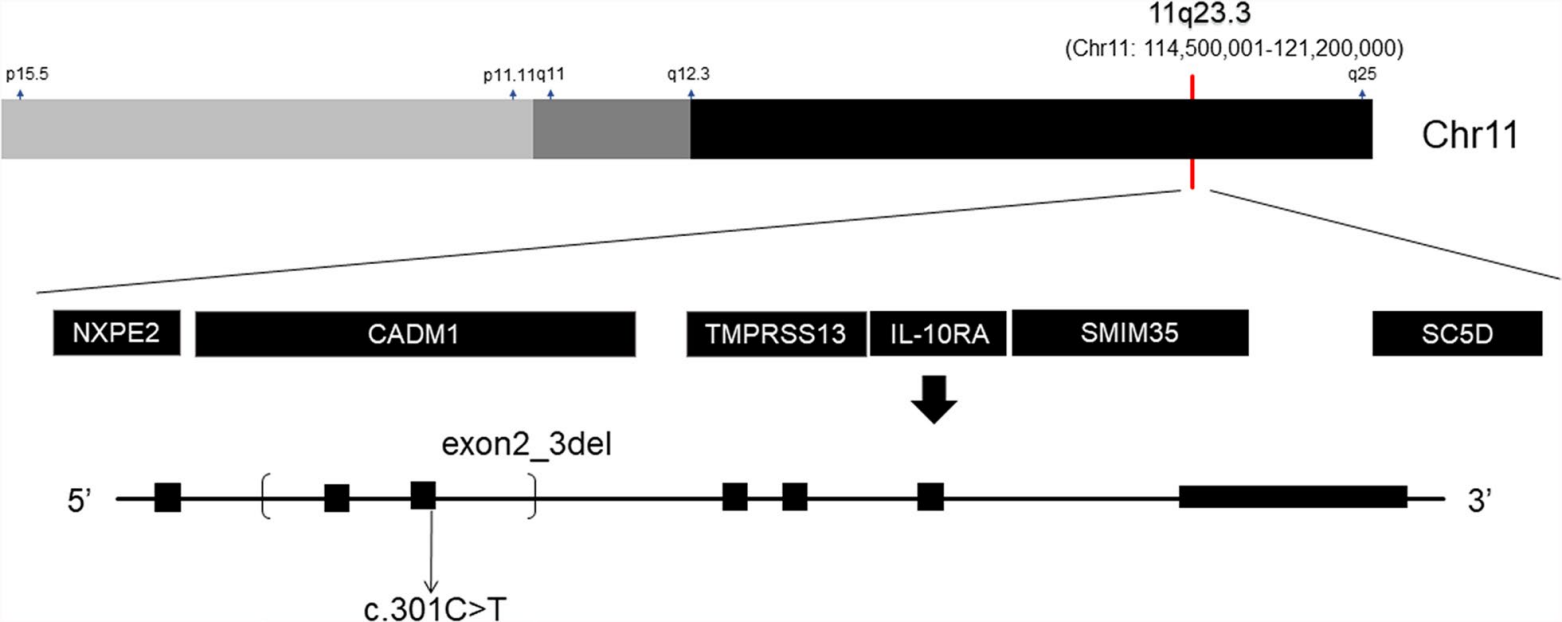
Positions of the variants among two cases. Light gray: short arm of chromosome 11; dark gray: long arm of chromosome 11; dark: chromosome 11 with 11q12.3 to 11q25 region

The two patients here seemed to be homozygosity of point mutation, which could not be verified by parental validation. Their classical clinical features, such as early onset age, severe perianal abscess in the infantile period and deep ulceration of the intestinal mucosal (see Table 1), diminish the possibility of a healthy carrier, leading to the inference of three possibilities [11, 23]: Firstly, the patient inherits gene deletion, which is hardly detected by general WES analysis, from one of the parents; Secondly, the patient develops the disease through UPD; Thirdly, the patient is not biologically related to his or her parents. Interestingly, the two cases reported here separately represent the former two circumstances, providing implications for genomic diagnosis (Fig. 5).

Patient 1 exhibited UPD, which refers to a condition in which both homologs of the chromosome are from the same parent. Based on whether two homologs are identical, UPD is classified into two types: heterodisomy (UPhD) and isodisomy (UPiD). The latter one could result in homozygosity for a recessive mutation, causing the offspring of a heterozygote and a wild type to present disease manifestation. This is the first UPD patient reported with *IL10RA* associated IBD. Part of his 11th chromosome was of paternal origin, possibly caused by post-zygotic mitotic recombination after normal fertilization [24]. The rate of partial UPD in the general population is approximately one per 3806 chromosome pairs [25]. Certain homozygosity mapping algorithms like SNPitty [26] or homozygous stretch identifier [27] are helpful to detect UPD utilizing WES data. However, we still performed the CGH array to validate the results since it is still the gold standard to test UPD according to the ACMG statement [28]. The diagnosis of UPD could inform parents of the fact that they are less likely to give birth to another ill child compared with the general situation of autosomal recessive disease because UPD is a rare event, thus reassuring them during genetic counselling.

Patient 2 carried a large fragment loss in exons 2 and 3. Although this variant is first reported, there are case reports on other kinds of large fragment deletions of *IL10RA* before (Table 2). The majority of patients carry one single point mutation accompanying with large fragment deletion in the other allele, which is hard to be detected by regular WES analysis, suggesting that we should be cautious about making genomic diagnosis facing patients with only monoallelic mutation or Sanger validation contradicting to Mendel’s law. The gene deletion and other structural variations could be inferred by WES through analyzing mapping discordance between a sample read and the reference genome [10]. Besides, whole-genomic sequencing (WGS) and CGH could also serve as complementary tools to detect deletions [10].

As HSCT is the only curable treatment for this disease [7], it is recommended for both patients. During the disease course, patient 1 was treated with mesalamine (250 mg each day), thalidomide (12.5 mg each day) and discharged after partially symptomatic remission. HSCT was performed at the age of 6 months and the patient recovered well after surgery. Patient 2 was treated with mesalamine (250 mg each day), thalidomide (8 mg each day), and antibiotics. She also underwent protective enterostomy because of severe intestinal diseases. She presented high fever post-operation. Metagenomic next-generation sequencing identified 7 unique reads of Candida parapsilosis in her blood, and blood culture confirmed the results. She was transferred to the PICU and was stable after receiving administration of voriconazole and caspofungin and other supportive treatments. In June of 2022, she received HSCT and the efficacy was well.

## Conclusions

We identified two novel variants expand the mutation spectrum of *IL10RA* related IBD. It is noteworthy that these special cases emphasize the importance of considering the possibility of gene deletion and UPD when analyzing apparent homozygosity, especially when the patient, from a nonconsanguineous family, carries a nonfounder mutation.

## Data Availability

The data that support the findings of this study are available from the authors but restrictions apply to the availability of these data, which were used under license from the Ethics Committee of the Children’s Hospital of Fudan University for the current study, and so are not publicly available. Data are, however, available from the authors upon reasonable request and with permission from the Children’s Hospital of Fudan University.
